# Supplementary material for: Impact of transposable elements on genome structure and evolution in bread wheat
Source: Genome Biol. 2018 Aug 17;19:103. doi: 10.1186/s13059-018-1479-0 (PMC6097303; doi:10.1186/s13059-018-1479-0)
Supplement: Supplementary file 1 — Tables S1 to S2, Figures S1 to S20. (PDF 2879 kb) [file 13059_2018_1479_MOESM1_ESM.pdf]

Supplementary Materials for

Impact of transposable elements on genome structure and evolution in bread wheat

Thomas Wicker[1†], Heidrun Gundlach[2†], Manuel Spannagl[2], Cristobal Uauy[3], Philippa Borrill[3], Ricardo H. Ramírez-González[3], Romain De Oliveira[4], International Wheat Genome Sequencing Consortium, Klaus F. X. Mayer[2], Etienne Paux[4], Frédéric Choulet[4]*

correspondence to: frederic.choulet@inra.fr

This PDF file includes:

Tables S1 to S2
Figures S1 to S20

Table S1: Metrics of the wheat full length LTR-retrotransposon (flLTR-RT) complement. 'U' denotes the unassigned assembly portion

		number	number per Mb	RLC / RLG ratio	Mb	median length (bps)	LTR % of length*	median age (Myrs)	median 20-mer frequency
ABD	all	112,744	7.9		1,080.6	9,584		1.18	10,791
ABD	RLC	58,690	4.1	2.1	522.9	8,536	15.0	0.95	16,720
ABD	RLG	28,489	2.0		325.1	10,436	7.1	1.30	3,055
ABD	RLX	25,565	1.8		232.6	8,091	9.2	1.66	5,102
A	all	40,328	8.3		388.3	9,629		1.23	10,750
A	RLC	20,636	4.2	1.9	183.6	8,552	15.3	0.98	16,614
A	RLG	10,835	2.2		124.0	10,473	7.2	1.34	3,343
A	RLX	8,857	1.8		80.7	8,091	8.9	1.73	5,273
B	all	39,859	7.8		384.4	9,645		1.27	9,640
B	RLC	20,367	4.0	2.0	183.3	8,561	14.7	1.04	15,243
B	RLG	10,328	2.0		118.9	10,594	6.4	1.36	2,750
B	RLX	9,164	1.8		82.3	8,079	9.2	1.72	5,174
D	all	31,055	8.0		291.0	9,369		1.02	12,379
D	RLC	17,021	4.4	2.4	149.2	8,447	14.9	0.83	18,928
D	RLG	7,044	1.8		78.8	10,270	8.6	1.14	2,913
D	RLX	6,990	1.8		62.9	8,065	9.9	1.48	4,515
U	all	1,502	3.5		16.9	11,241		0.94	9,624
U	RLC	666	1.5	2.4	6.8	8,584	12.2	0.90	14,901
U	RLG	282	0.7		3.5	11,074	6.7	0.84	4,570
U	RLX	554	1.3		6.7	10,267	6.2	1.08	6,613

* length percent of one terminal repeat in relation to the element length

Table S2: Coordinates of the chromosome compartments defined based on structural and functional features in [24]. R1 and R3: distal regions of short and long chromosome arms, respectively. R2a and R2b: interstitial regions on short and long chromosome arms, respectively. C: centromeric/pericentromeric regions. Positions are given in Mb.

	Length (Mb)	R1/R2a boundary	R2a/C boundary	C/R2b boundary	R2b/R3 boundary	Centromere
chr1A	594	59	151	231	480	213.5
chr1B	689	62	172	277	534	236.7
chr1D	495	29	98	171	385	172.5
chr2A	780	42	206	379	662	340.0
chr2B	801	59	248	433	660	349.4
chr2D	651	37	192	338	520	268.0
chr3A	750	62	249	414	670	319.0
chr3B	830	66	257	407	728	346.8
chr3D	615	49	167	287	543	242.7
chr4A	744	41	180	414	594	265.5
chr4B	673	42	186	360	537	319.3
chr4D	509	10	135	288	432	185.8
chr5A	709	39	140	260	427	253.8
chr5B	713	52	140	221	430	198.9
chr5D	565	46	128	207	345	188.8
chr6A	617	46	216	409	556	285.3
chr6B	720	56	221	429	651	325.2
chr6D	473	44	164	280	410	214.1
chr7A	736	89	239	416	659	359.4
chr7B	750	12	146	418	660	296.4
chr7D	638	84	200	373	552	339.4

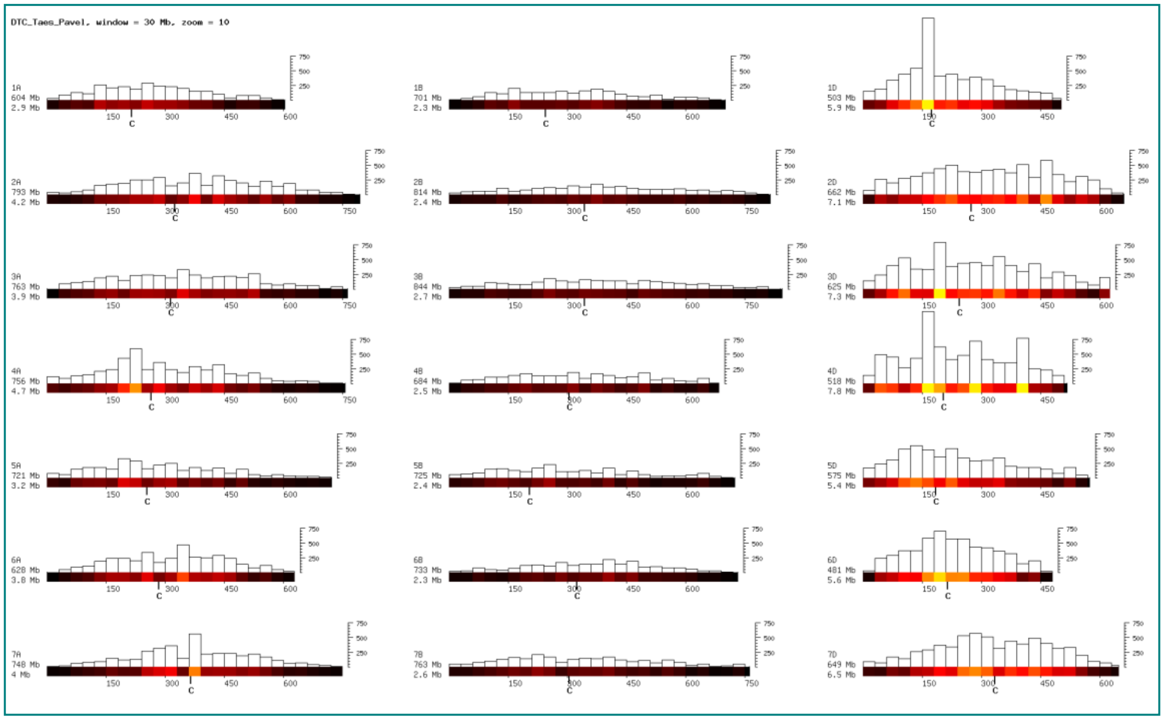

Figure S1: Distribution of the DTC_famc10.3 (Pavel) subfamily along wheat chromosomes. Pavel is more abundant in the D genome than in the A and B genomes, suggesting it underwent a burst of activity after the D genome diverged from the other two. The TE distribution is shown in 30 Mb windows along chromosomes. TE abundance per 30 Mb-window is shown as heat map and as a bar plot. The x-axis indicates the physical position in Mb, while the y-axis indicates the number of kb the TE family contributes to each 30 Mb. The label to the left of each chromosome indicates the chromosome name, chromosome size in Mb, and total contribution of the TE family to the chromosome sequence (in Mb).

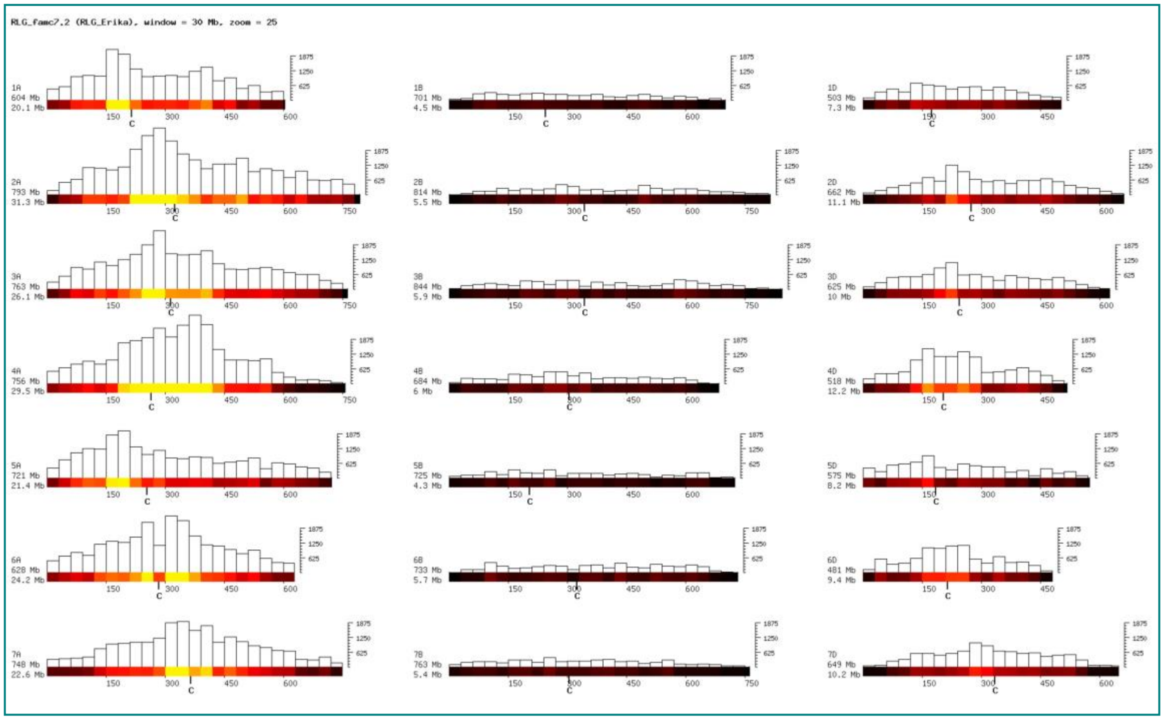

Figure S2: Distribution of the RLG_famc7.2 (Erika) subfamily along wheat chromosomes. Erika is most abundant in the A genome and somewhat less in the D genome, and it is the only subfamily that is depleted in the D genome. TE abundance per 30 Mb window is shown as heat map and as a bar plot. The x-axis indicates the physical position in Mb, while the y-axis indicates the number of kb the TE family contributes to each 30 Mb. The label to the left of each chromosome indicates the chromosome name, chromosome size in Mb, and total contribution of the TE family to the chromosome sequence (in Mb).

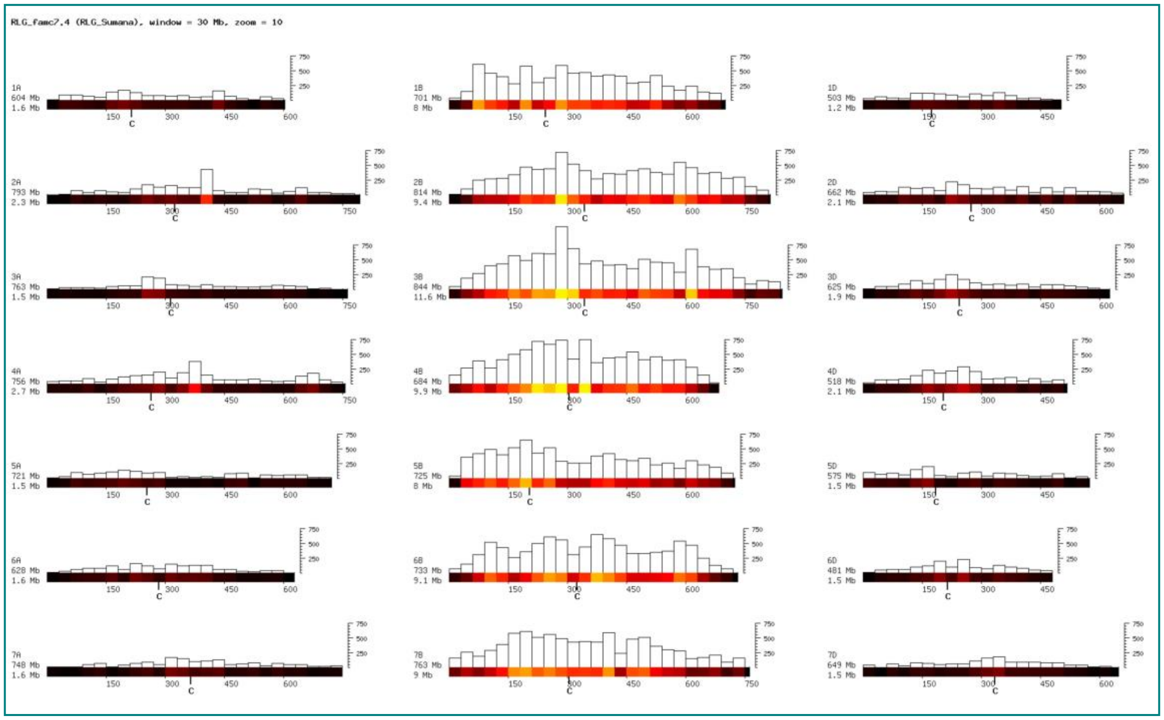

Figure S3: Distribution of the RLG_famc7.4 (Sumana) subfamily along wheat chromosomes. Sumana is more abundant in the B genome. TE abundance per 30 Mb window is shown as heat map and as a bar plot. The x-axis indicates the physical position in Mb, while the y-axis indicates the number of kb the TE family contributes to each 30 Mb. The label to the left of each chromosome indicates the chromosome name, chromosome size in Mb, and total contribution of the TE family to the chromosome sequence (in Mb).

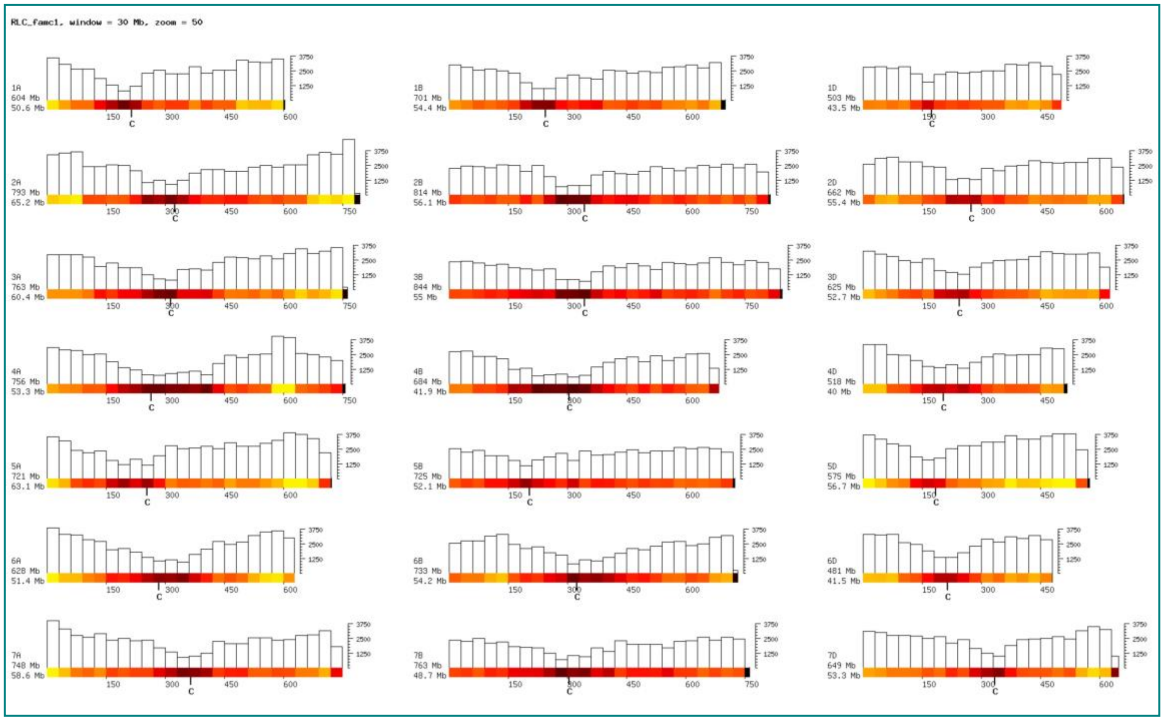

Figure S4: Distribution of the RLC_famc1 (Angela) family along wheat chromosomes. Angela is generally enriched toward centromeres and depleted in central regions of chromosomes. The TE distribution is shown in 30 Mb windows along chromosomes. TE abundance per 30 Mb window is shown as heat map and as a bar plot. The x-axis indicates the physical position in Mb, while ty y-axis indicates the number of kb the TE family contributes to each 30 Mb. The label to the left of each chromosome indicates the chromosome name, chromosome size in Mb, and total contribution of the TE family to the chromosome sequence (in Mb).

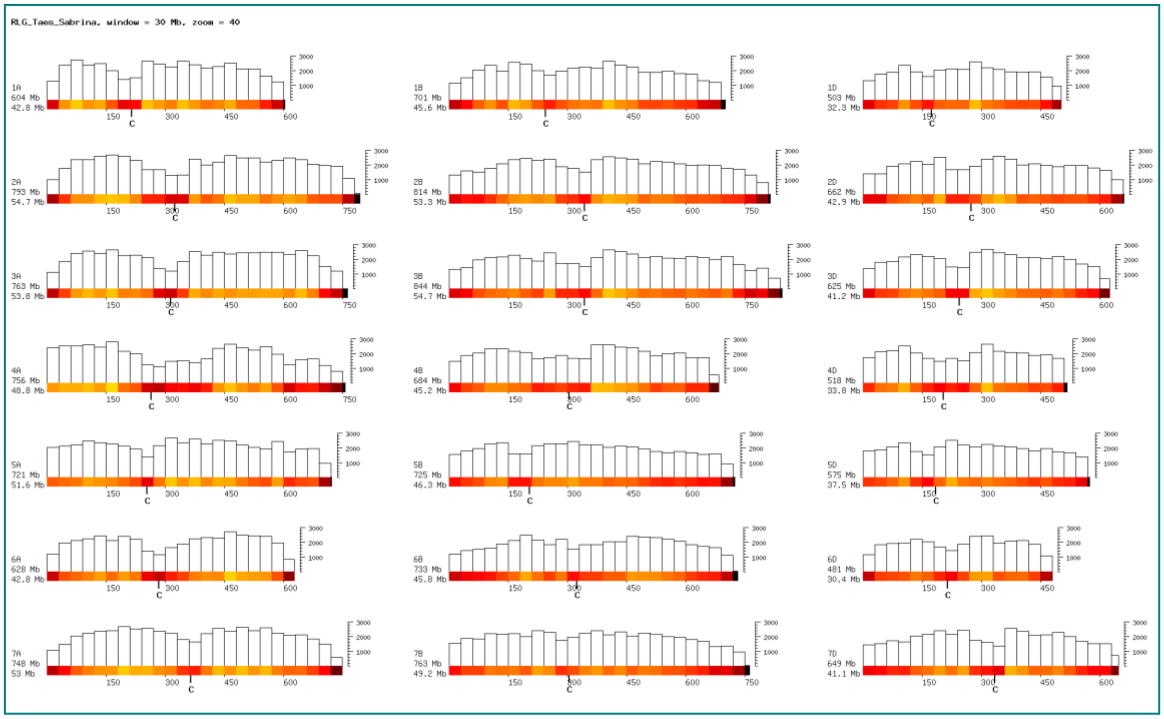

Figure S5: Distribution of the RLG_famc2 (Sabrina) family along wheat chromosomes. Sabrina is enriched in the central parts of chromosome arms and depleted in distal and proximal regions. The TE distribution is shown in 30 Mb windows along chromosomes. TE abundance per 30 Mb window is shown as heat map and as a bar plot. The x-axis indicates the physical position in Mb, while the y-axis indicates the number of kb the TE family contributes to each 30 Mb. The label to the left of each chromosome indicates the chromosome name, chromosome size in Mb, and total contribution of the TE family to the chromosome sequence (in Mb).

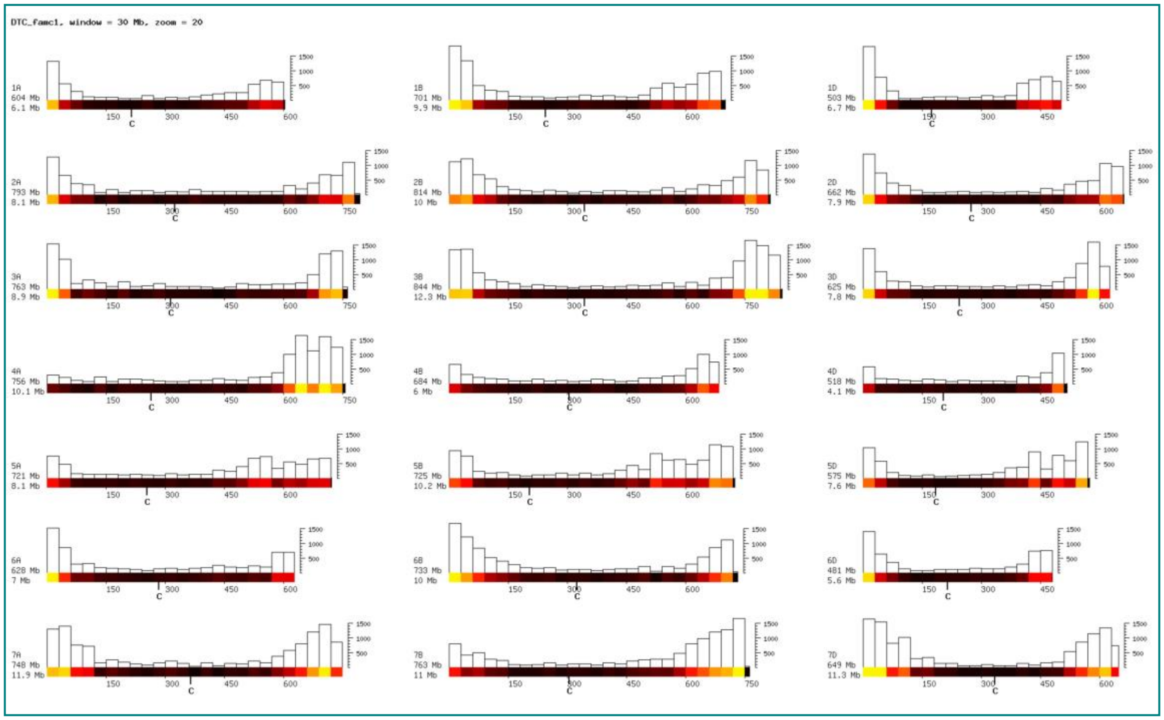

Figure S6: Distribution of the DTC_famc1 (Caspar) family along wheat chromosomes. Caspar is enriched in distal regions and practically absent from proximal regions. The TE distribution is shown in 30 Mb windows along chromosomes. TE abundance per 30 Mb window is shown as heat map and as a bar plot. The x-axis indicates the physical position in Mb, while the y-axis indicates the number of kb the TE family contributes to each 30 Mb. The label to the left of each chromosome indicates the chromosome name, chromosome size in Mb, and total contribution of the TE family to the chromosome sequence (in Mb).

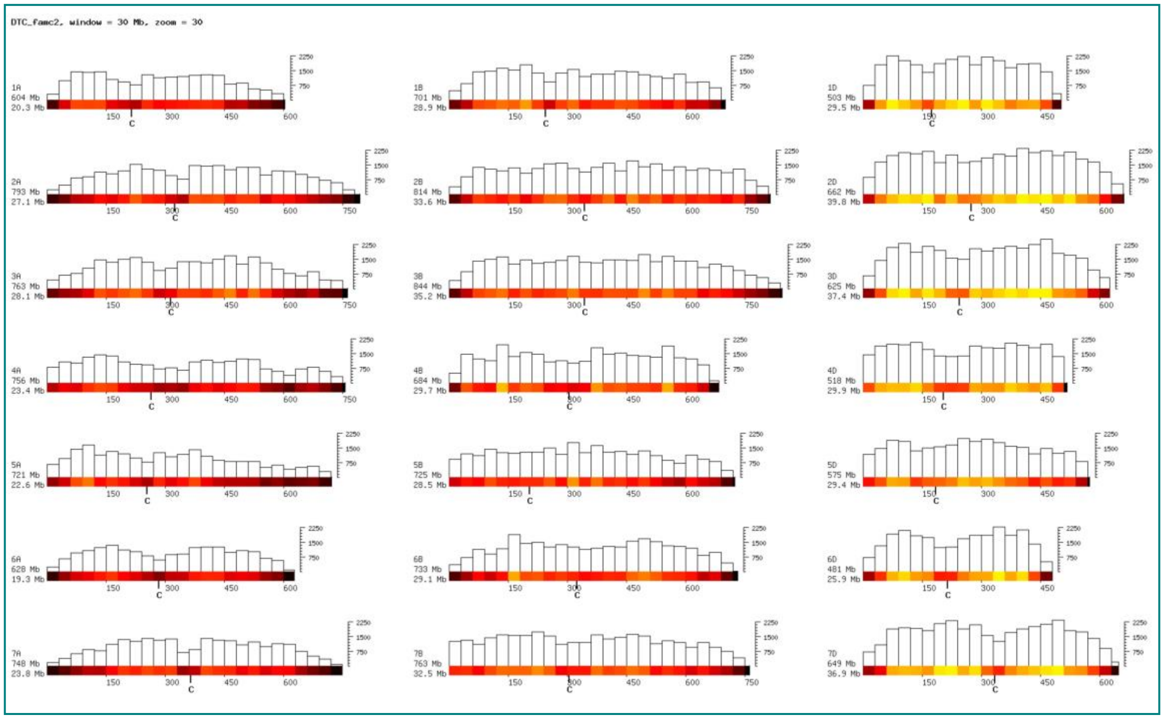

Figure S7: Distribution of the DTC_famc2 (Jorge) family along wheat chromosomes. Jorge is enriched in the central parts of chromosome arms and depleted in telomeric and centromeric regions. The TE distribution is shown in 30 Mb windows along chromosomes. TE abundance per 30 Mb window is shown as heat map and as a bar plot. The x-axis indicates the physical position in Mb, while the y-axis indicates the number of kb the TE family contributes to each 30 Mb. The label to the left of each chromosome indicates the chromosome name, chromosome size in Mb, and total contribution of the TE family to the chromosome sequence (in Mb).

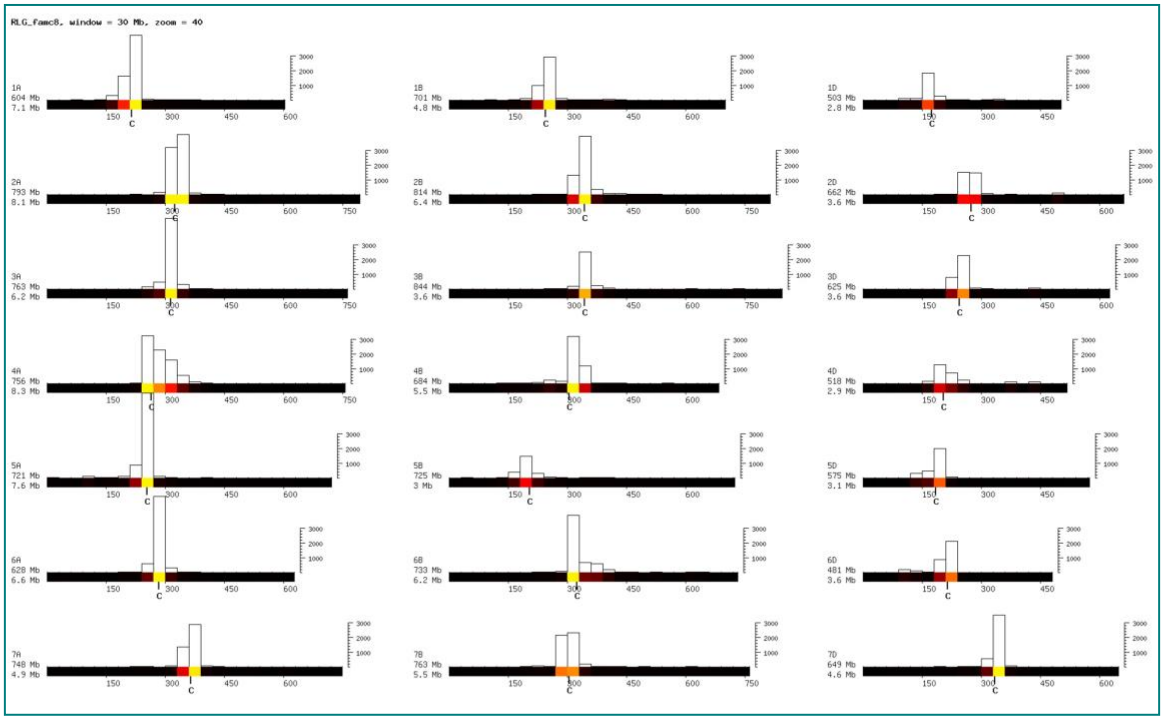

Figure S8: Distribution of RLG_famc8 (Cereba) retrotransposons along wheat chromosomes. Cereba is highy enriched in centromeric regions. The TE distribution is shown in 30 Mb windows along chromosomes. TE abundance per 30 Mb window is shown as heat map and as a bar plot. The x-axis indicates the physical position in Mb, while the y-axis indicates the number of kb the TE family contributes to each 30 Mb. The label to the left of each chromosome indicates the chromosome name, chromosome size in Mb, and total contribution of the TE family to the chromosome sequence (in Mb).

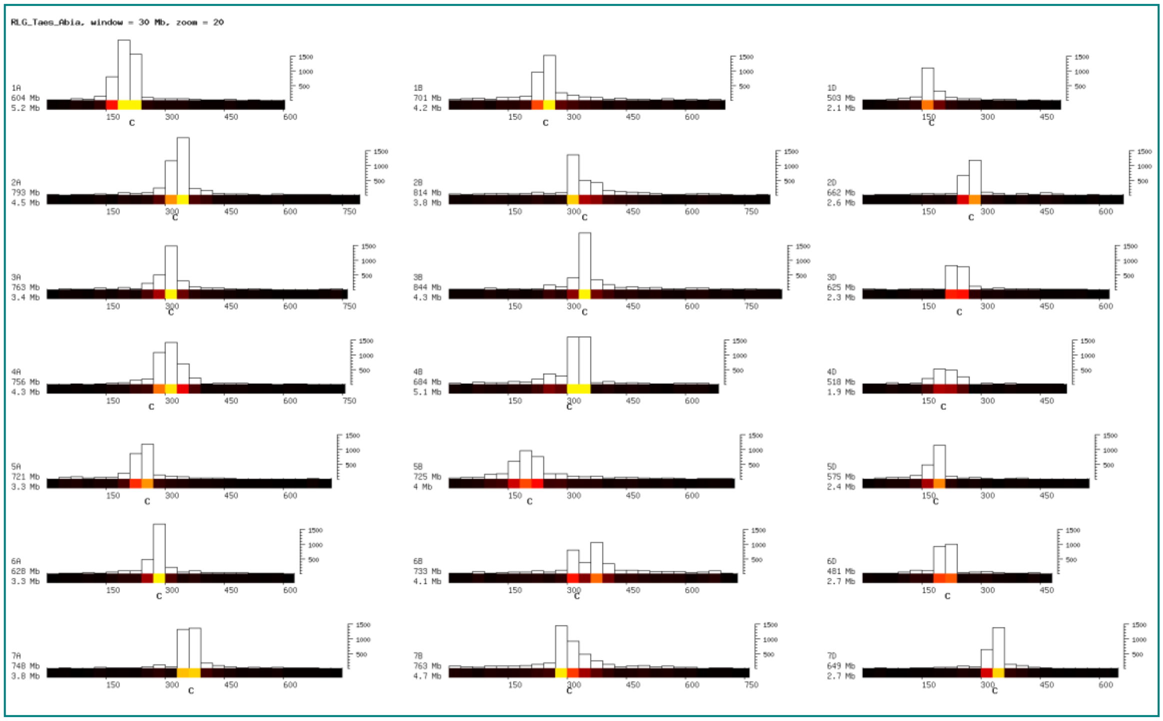

Figure S9: Distribution of RLG_famc39 (Abia) retrotransposons along wheat chromosomes. Abia is highy enriched in centromeric regions. The TE distribution is shown in 30 Mb windows along chromosomes. TE abundance per 30 Mb window is shown as heat map and as a bar plot. The x-axis indicates the physical position in Mb, while the y-axis indicates the number of kb the TE family contributes to each 30 Mb. The label to the left of each chromosome indicates the chromosome name, chromosome size in Mb, and total contribution of the TE family to the chromosome sequence (in Mb).

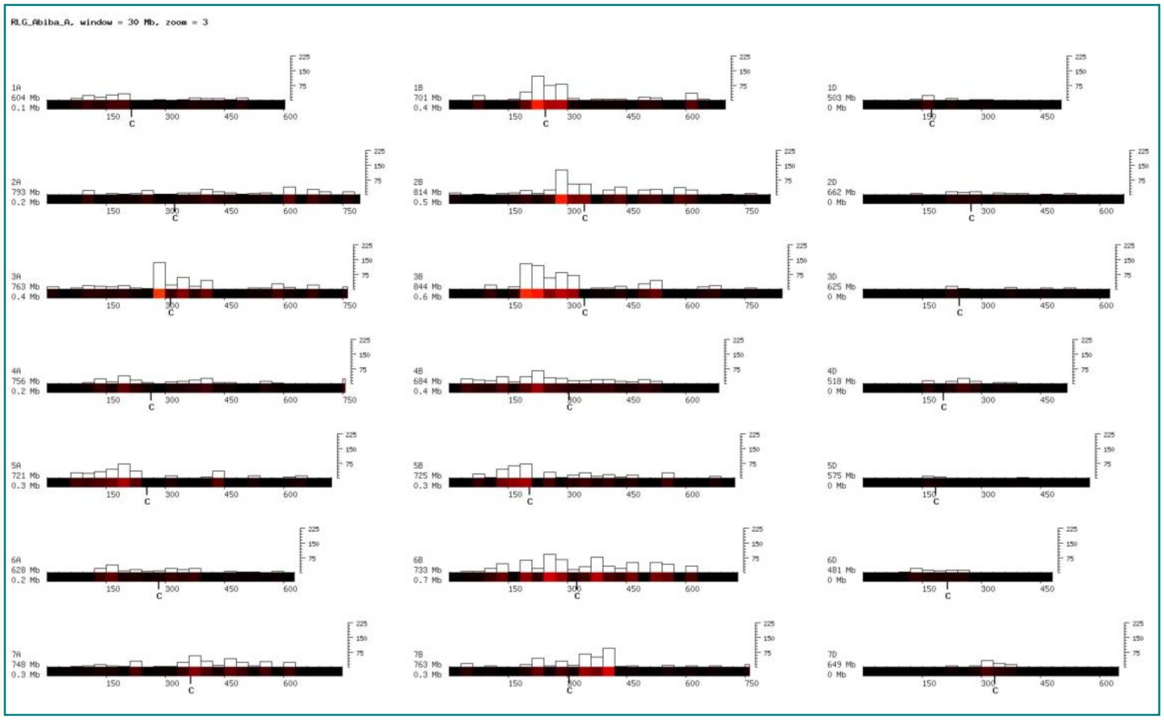

Figure S10: Distribution of RLG_famc40.1 (Abiba_A) subfamily along wheat chromosomes. The TE distribution is shown in 30 Mb windows along chromosomes. TE abundance per 30 Mb window is shown as heat map and as a bar plot. The x-axis indicates the physical position in Mb, while the y-axis indicates the number of kb the TE family contributes to each 30 Mb. The label to the left of each chromosome indicates the chromosome name, chromosome size in Mb, and total contribution of the TE family to the chromosome sequence (in Mb).

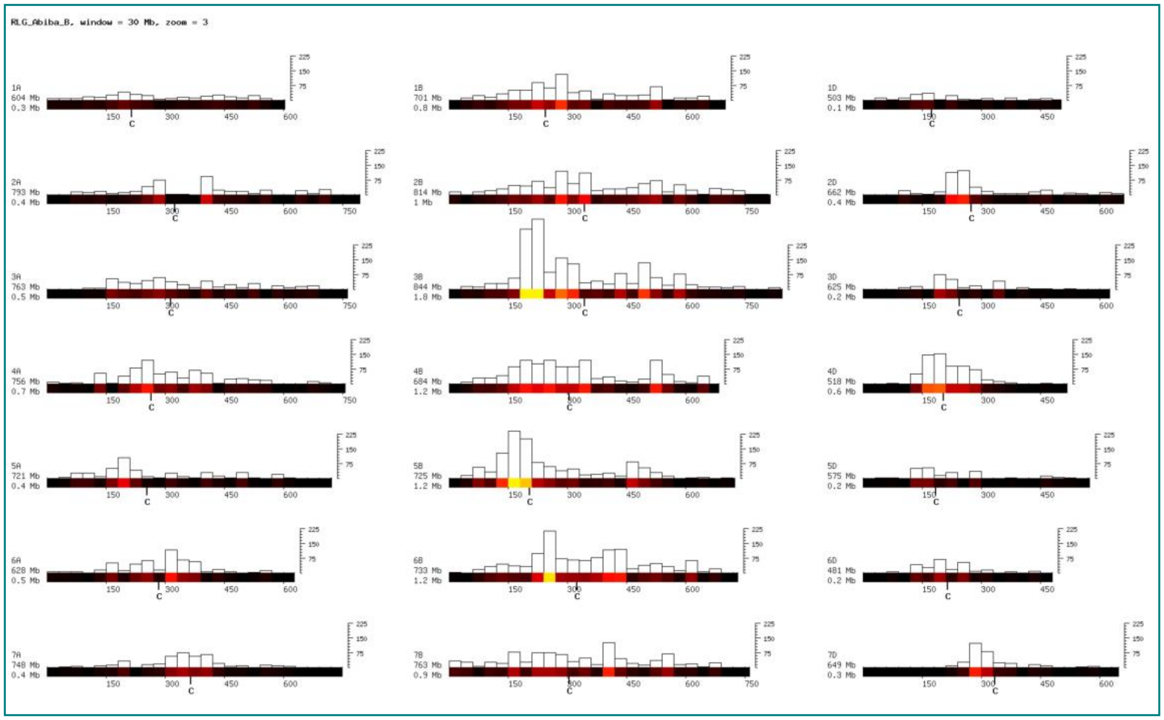

Figure S11: Distribution of RLG_famc40.2 (Abiba_B) subfamily along wheat chromosomes. The TE distribution is shown in 30 Mb windows along chromosomes. TE abundance per 30 Mb window is shown as heat map and as a bar plot. The x-axis indicates the physical position in Mb, while the y-axis indicates the number of kb the TE family contributes to each 30 Mb. The label to the left of each chromosome indicates the chromosome name, chromosome size in Mb, and total contribution of the TE family to the chromosome sequence (in Mb).

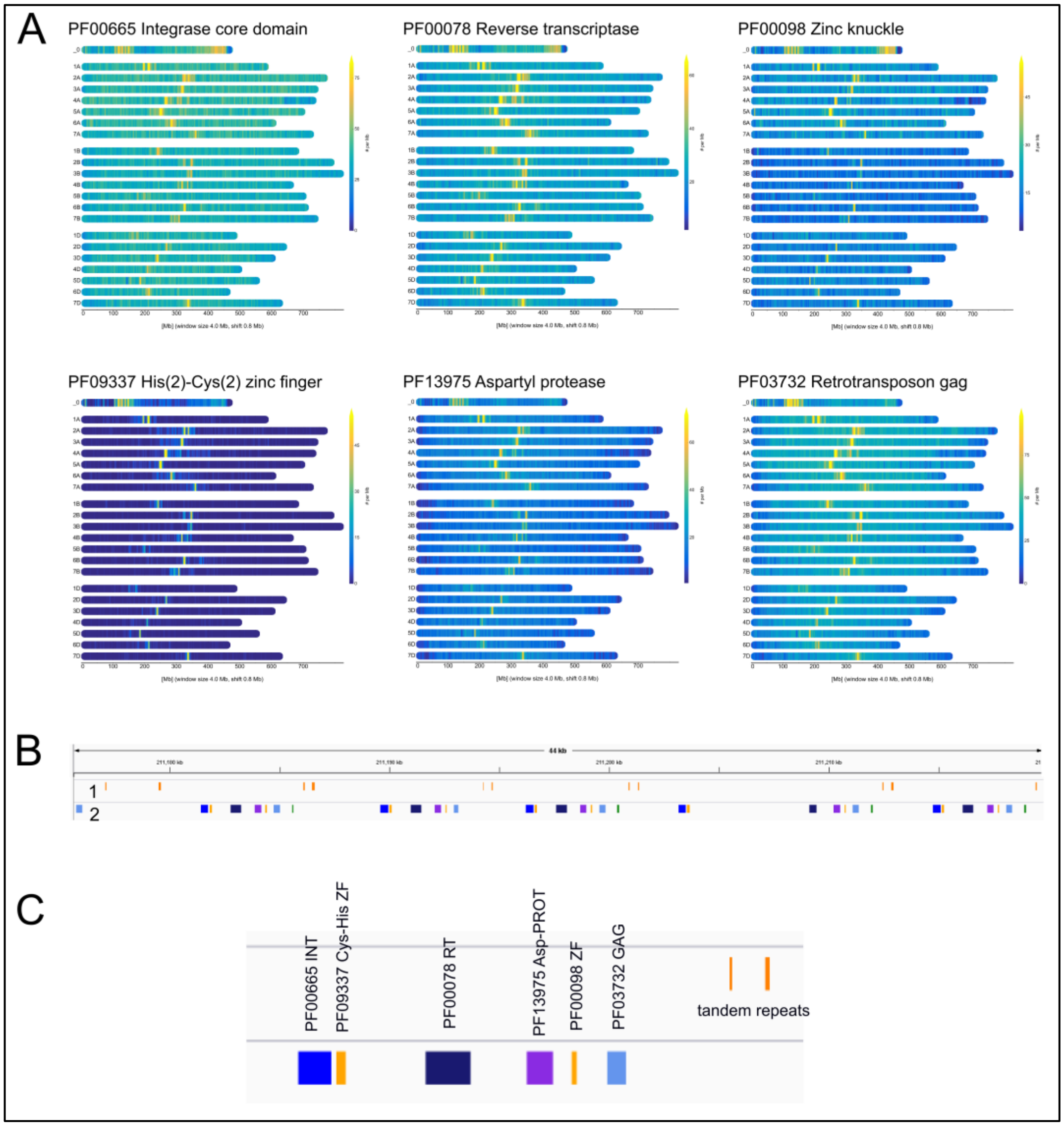

Figure S12: Location and structure of wheat centromeres. A. Transposon-related Pfam domains as centromere indicators. We identified six transposon-related Pfam domains that are strongly enriched in the centromeres. The PF09337 zinc-finger occurs even almost exclusively in the centromeres. Large amounts of them were found in the scaffolds unassigned to pseudomolecules (labeled "_0" at the top) showing that such repeated arrays were difficult to assemble. This may largely explain the differences observed between chromosomes in their centromere dimensions (strength or multiple hotspots) **B.** Zoomed in view on a 44 kb centromeric region of chromosome 4A showing tandemly repeated clusters of Cereba-associated Pfam domains (track 2). **C.** Zoomed in view on a centromere repeat unit comprising 6 domains and 2 short simple sequence repeats.

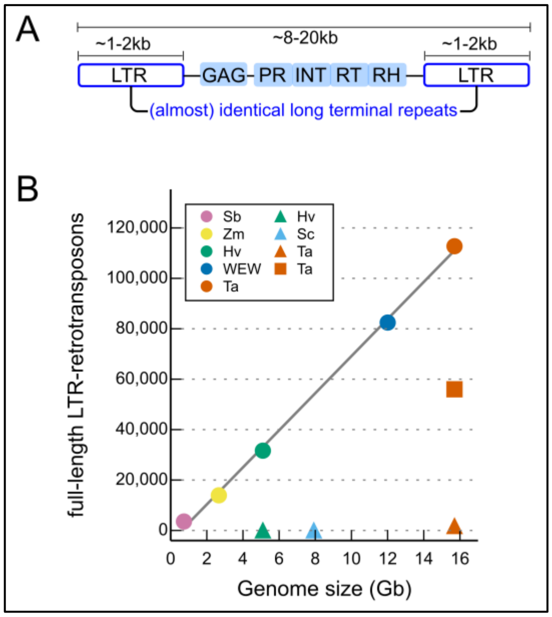

Figure S13: Full length LTR-retrotransposons and assembly quality. A. Schematic structure of a full length LTR-retrotransposon (flLTR-RT). **B.** Number of retrieved flLTR-RTs in different genome assemblies. The (almost) identical 1-2 kb long terminal repeats of flLTR-RTs were often not correctly reconstructed in previous contig assemblies (triangles). We observed a linear correlation between flLTR-RTs and genome size in high quality assemblies. The number of retrievable flLTR-RTs can thus serve as a metric to estimate the quality of the assembly of the repeated part of the genomes. Circles denote more complete assemblies, triangles lower quality contig assemblies. Sb: *Sorghum bicolor* [9]; Zm: *Zea mays* [10]; Hv: *Hordeum vulgare*, triangle [6], circle [23]; Sc: *Seccale cereale* [47]; WEW: wild emmer wheat [48]; Ta: bread wheat, triangle IWGSC-2014 [30], square TGAC_v1 [29] (missing mainly young Copia elements), circle IWGSC RefSeq_v1.0 from this study.

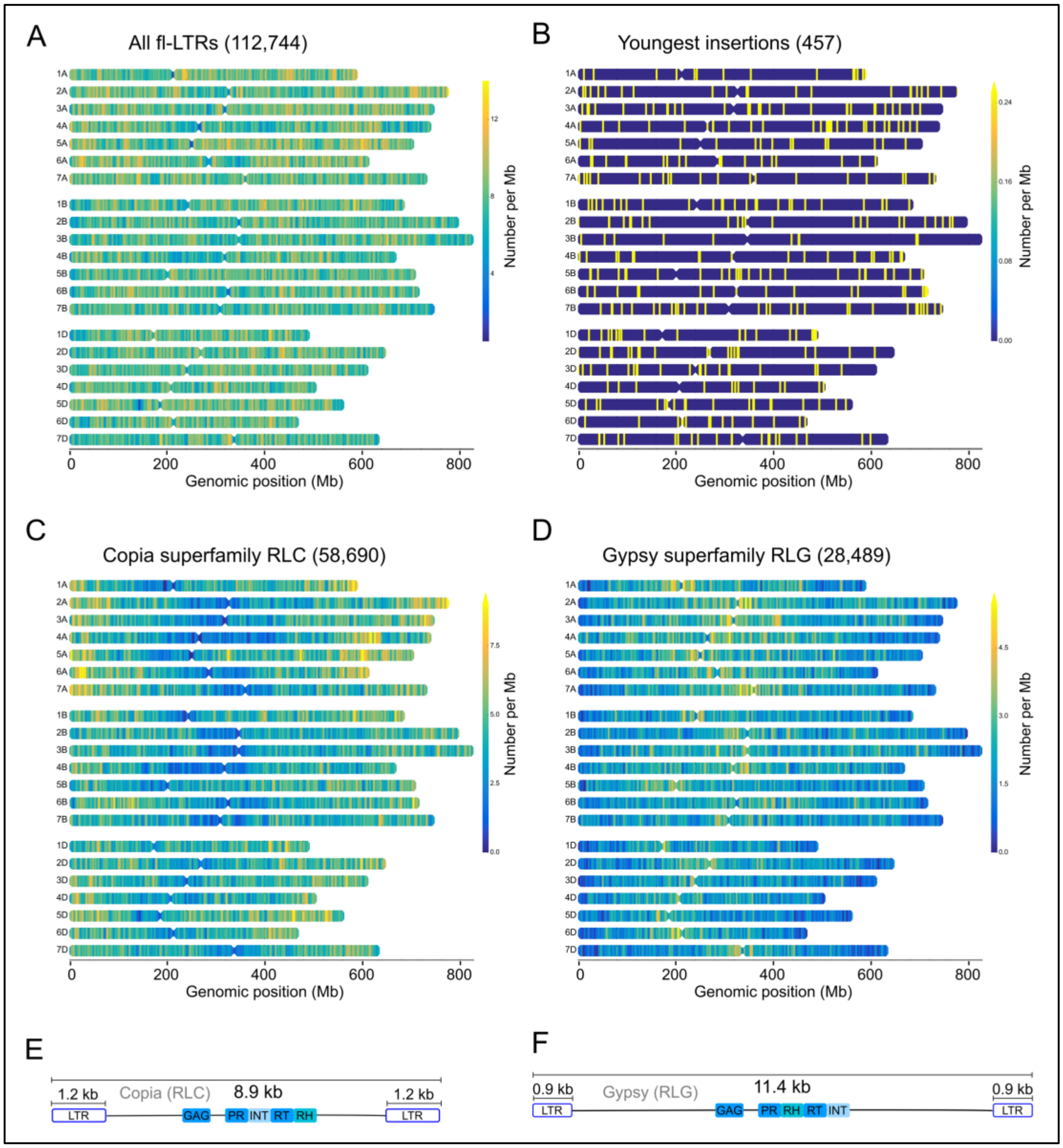

Figure S14: Chromosomal distribution of full length LTR-retrotransposons. A. All 112,744 insertion sites, **B.** Most recent 457 insertion sites from elements aged 0, RLCs . **C.** and **D.** Contrasting locations of the Copia (RLC) and Gypsy (RLG) superfamilies. **E.** and **F.** Copia and Gypsy element dimensions. The values correspond to median sizes over all RLC and RLG copies. Copia elements are shorter but have longer terminal repeats. The two terminal repeats cover around 30% of the Copia and only 14% of the Gypsy elements.

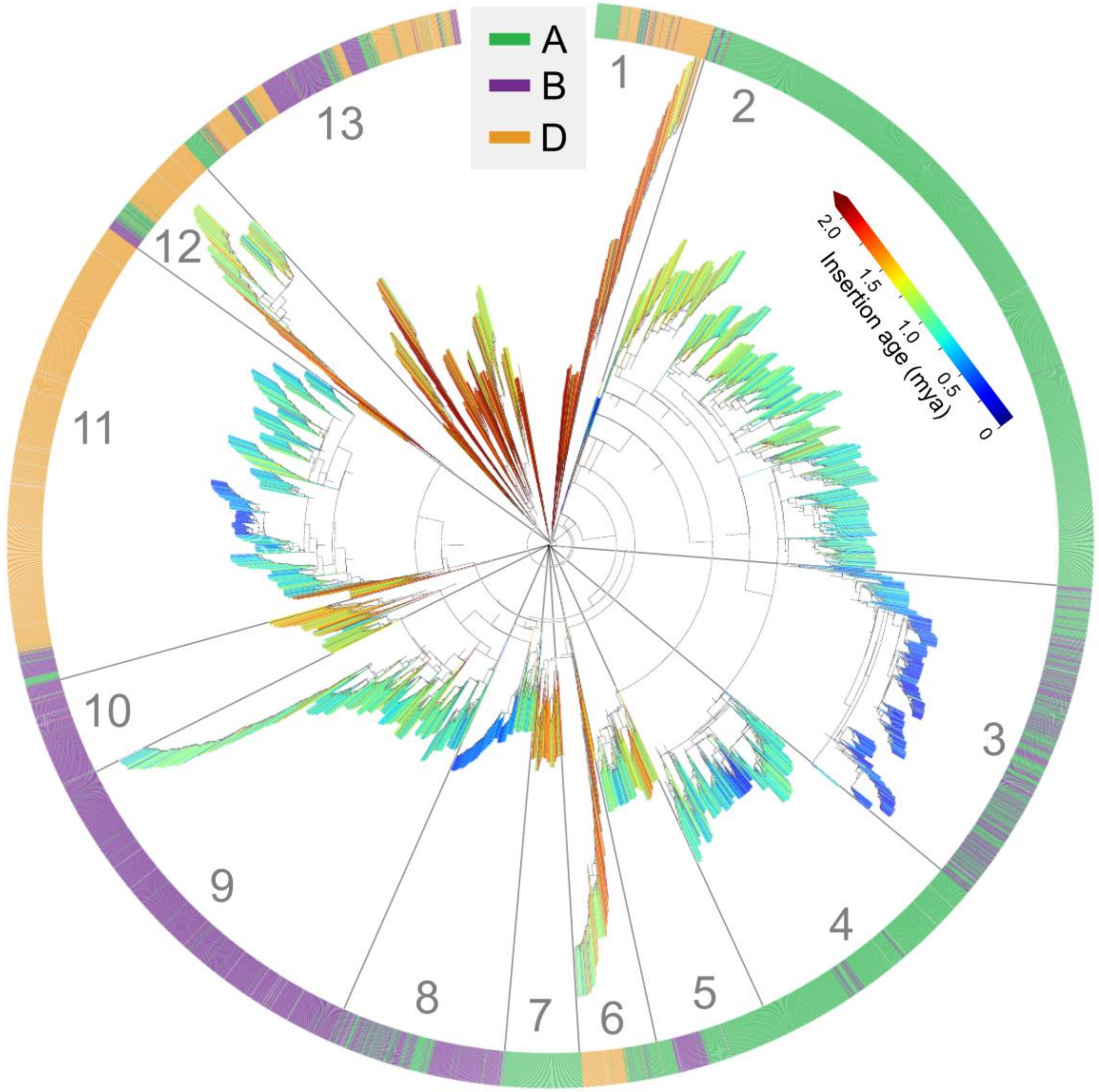

Figure S15: Phylogenetic tree for the largest flLTR-RT 90/90 cluster with 6,639 Copia members. The tree leaves are color coded by insertion age, the outer ring represents the subgenome localization of each element. Section lines and numbers were added manually to mark distinct constellations. Summary for Fig S15, S16 and S17: the recent proliferation in the AB tetraploid led to small scaled AB interweaving patterns in the outer ring (green-magenta) and always coincides with young age (blue color). The founder elements for these lineages came from either the A (e.g. Fig. S15-3, S16-9) or B (e.g. Fig. S16-2/4, S17-4) diploid ancestor. The large A (e.g. Fig. S15-2, S16-8) and B (e.g. Fig. S15-9, S16-5) sections contain mostly medium aged elements (1-1.5 Myrs) or, in the case of D, (e.g. Fig. S16-11) also younger lineages relating to the more recent Copia amplifications in the diploid. The oldest (~2 Myrs) and smallest lineages are located near the tree root and reveal several constellations where old elements seem to

have been transferred from either an A lineage (e.g. Fig. S15-1/6, S16-1, S17-3) or an B lineage (Fig. S15-11) to the D ancestor and gave rise to subsequent amplification rounds in D (see Fig 5, marked by an asterisk). We did not detect any transfer originating from D. Moreover, the D lineages were absent from primary branches at the root and all have an A or B precursor, consistent with the scenario of an homoploid hybridization between A and B at the origin of the D subgenome [34].

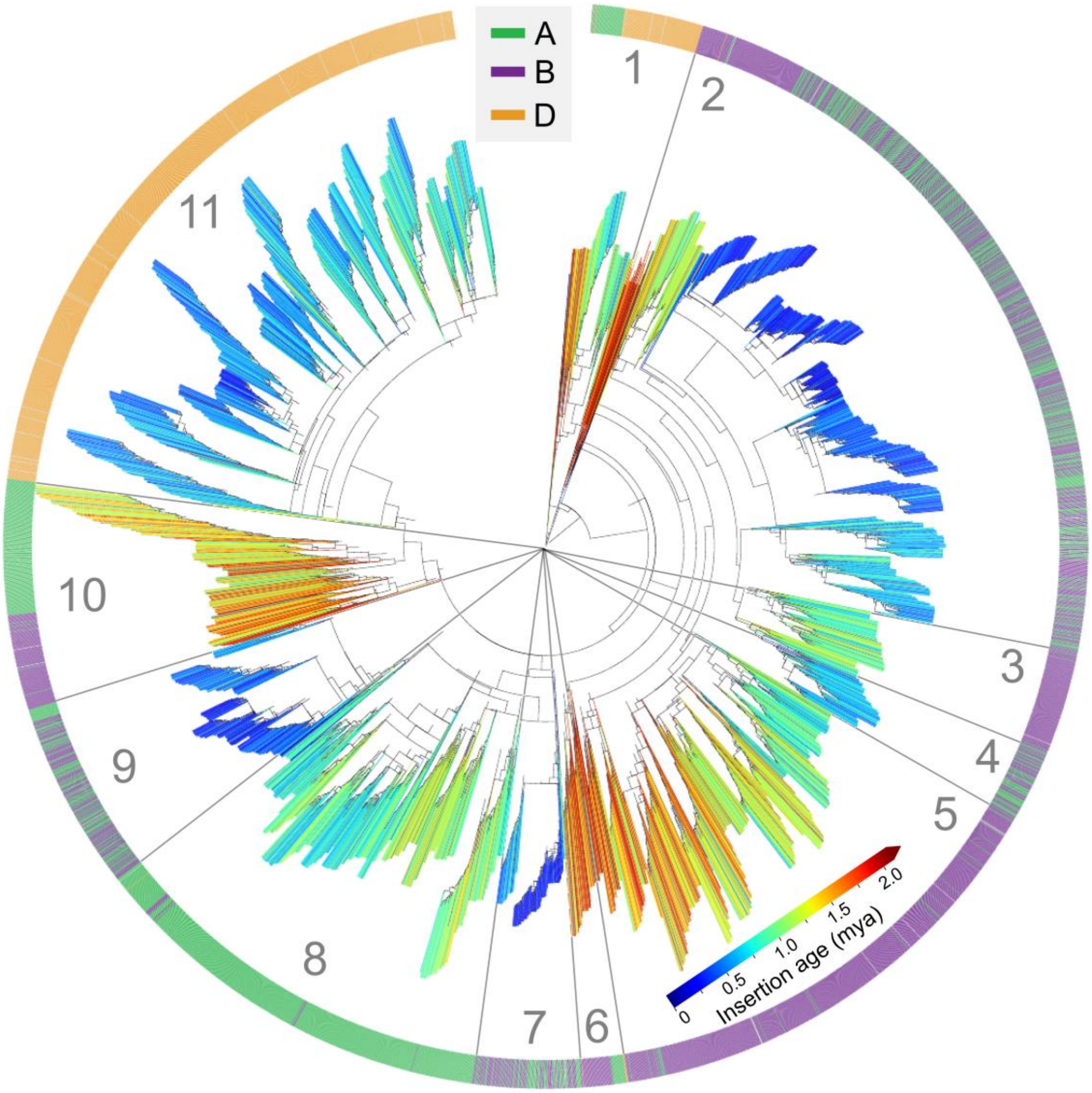

Figure S16: Phylogenetic tree for the second largest flLTR-RT 90/90 cluster with 5,387 Copia members. The tree leaves are color coded by insertion age, the outer ring represents the subgenome localization of each element. Section lines and numbers were added manually to mark distinct constellations.

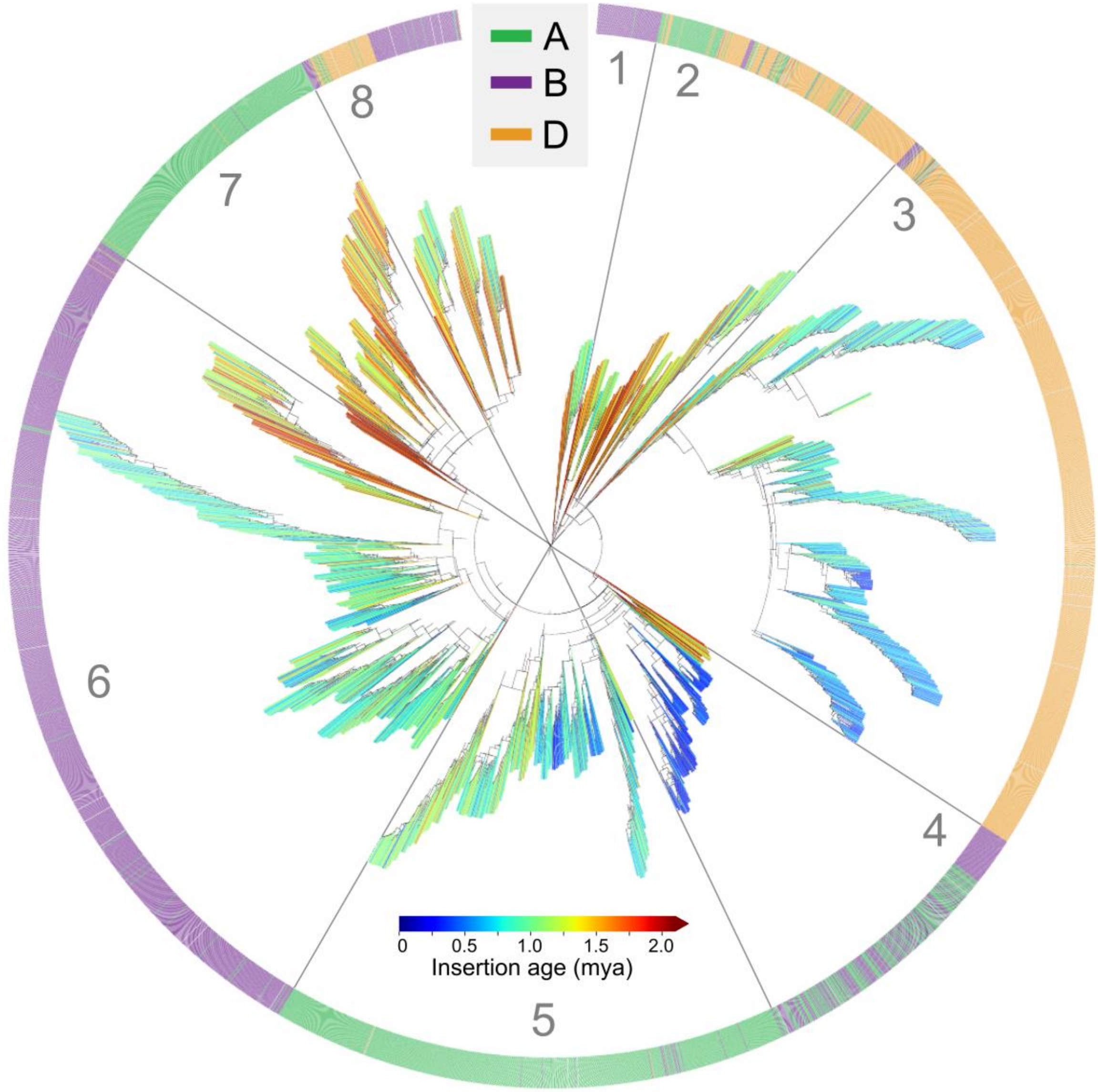

Figure S17: Phylogenetic tree for the third largest flLTR-RT 90/90 cluster with 4,564 Copia members. The tree leaves are color coded by insertion age, the outer ring represents the subgenome localization of each element. Section lines and numbers were added manually to mark distinct constellations.

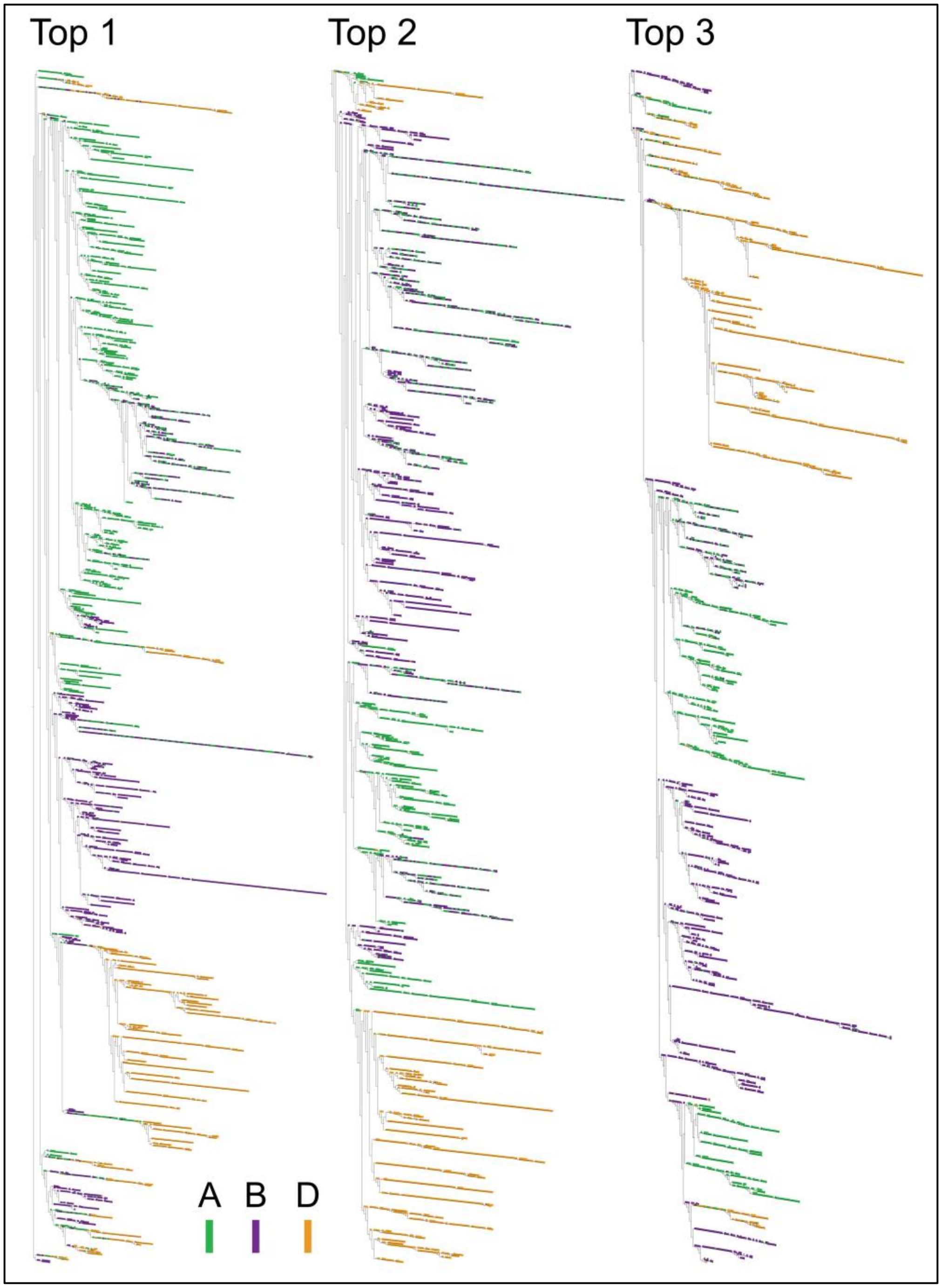

Figure S18: Tree topologies of the top3 90/90 clusters. The trees are depicted with identical branch lengths to get a better impression of their topologies.

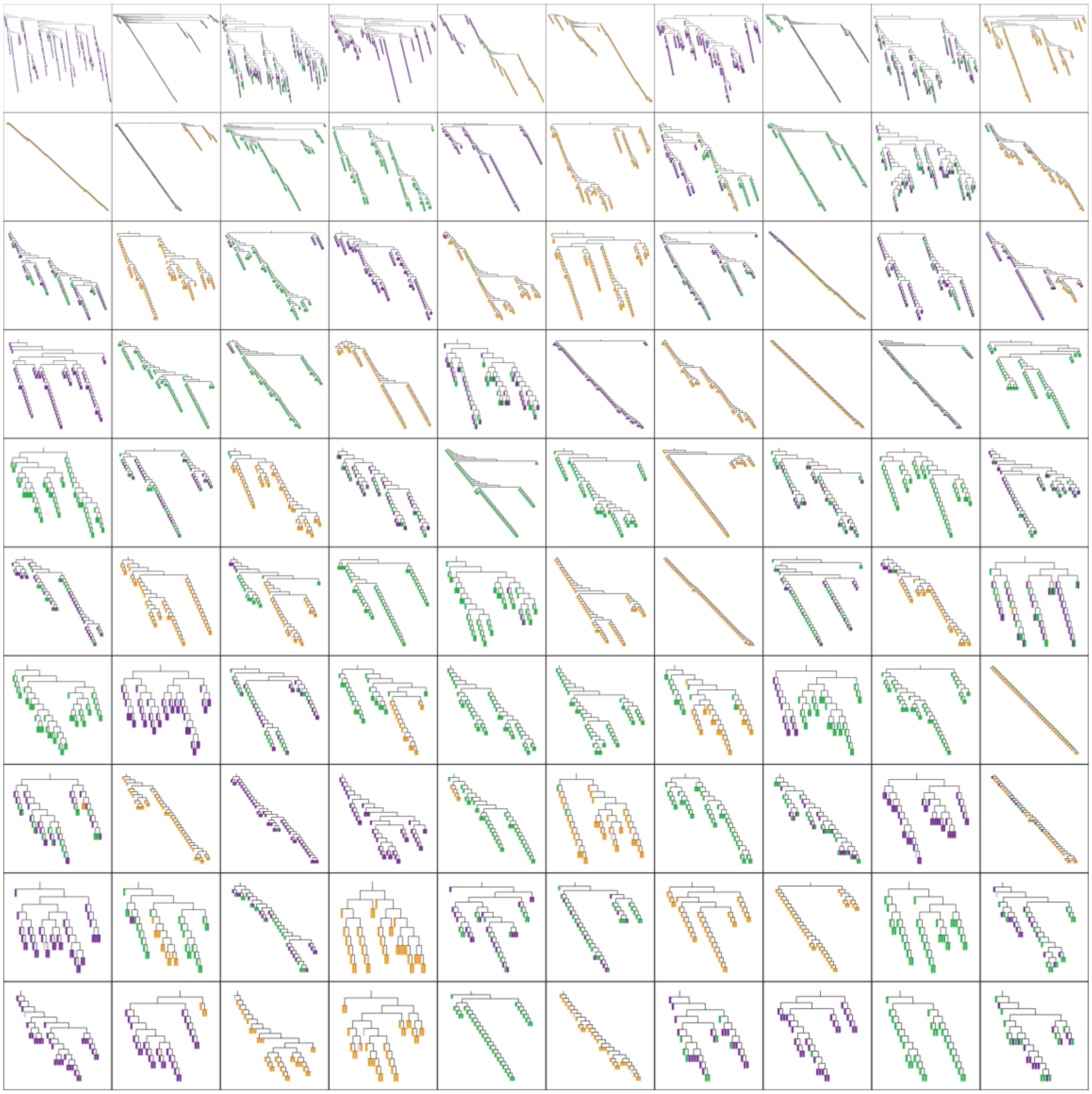

Figure S19: Tree topologies of the top4 to top103 90/90 clusters. Subgenome colors: A-green, B-magenta, D-orange.

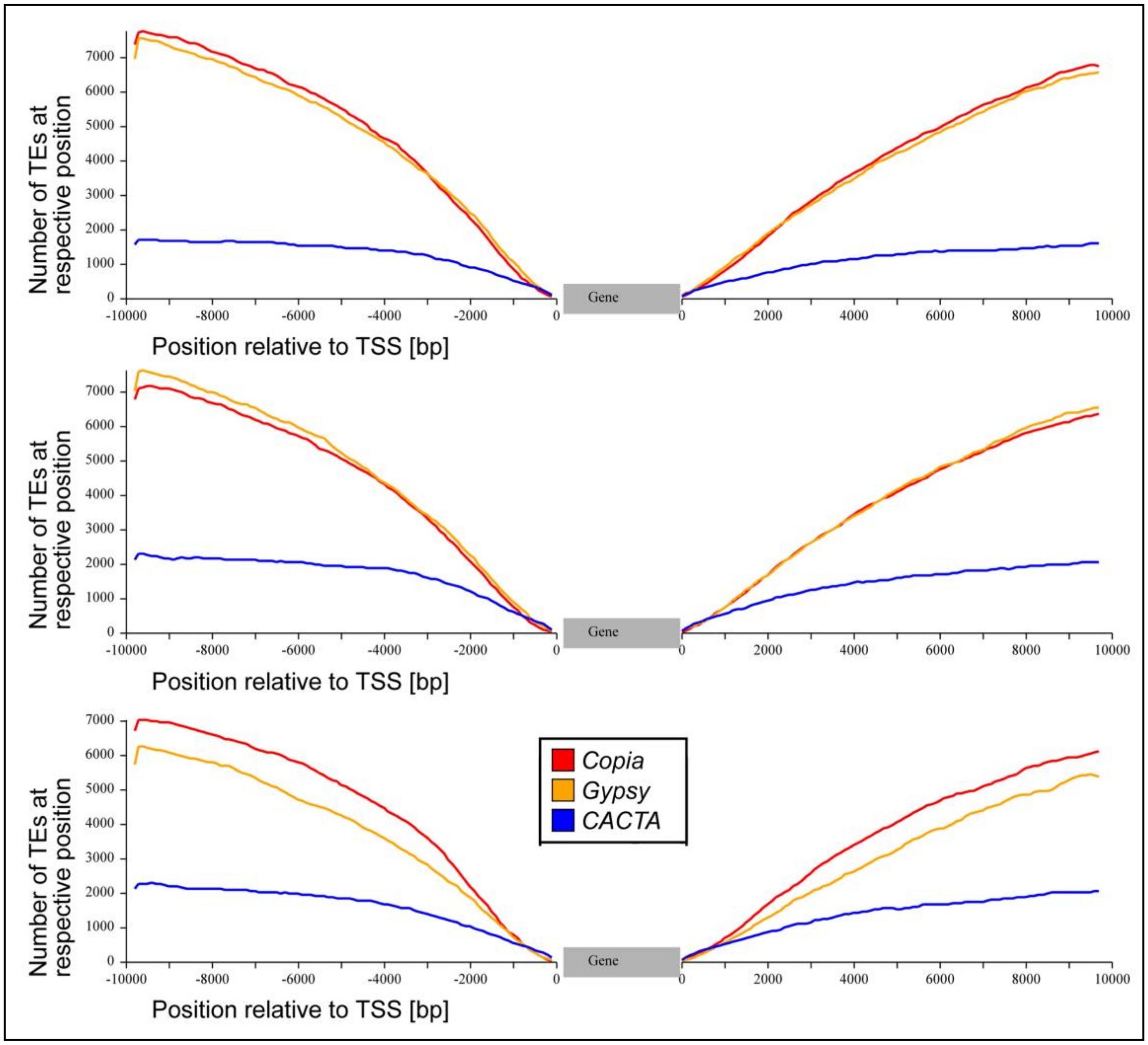

Figure S20: TE landscape surrounding genes. Genes from the three subgenomes were treated separately. For all genes, the 10 kb upstream of the transcription start site (TSS) and 10 kb downstream of the transcription end site were analyzed. Abundance of the different TE families was compiled for all genes of each subgenome. The plots include only those superfamilies that are highly abundant in intergenic regions. Note that the scale of the y-axis differs from that on Figure 7.
