# Supplementary material for: Impact of transposable elements on genome structure and evolution in bread wheat
Source: Genome Biol. 2018 Aug 17;19:103. doi: 10.1186/s13059-018-1479-0 (PMC6097303; doi:10.1186/s13059-018-1479-0)
Supplement: Supplementary file 2 — Reviewer reports and Author’s response to reviewers. (DOCX 30 kb) [file 13059_2018_1479_MOESM2_ESM.docx]

Reviewer reports and Author’s response to reviewers

First round of Review:

**Reviewer 1:**

**Are the methods appropriate to the aims of the study, are they well described, and are necessary controls included?**

In general yes, but see the review.

**Are the conclusions adequately supported by the data shown?**

No. In my opinion there are many unsupported conclusions raised from correlations that the authors transform in causal relationships.

**Are sufficient details provided to allow replication and comparison with related analyses that may have been performed?**

In general yes.

**Does the work represent a significant advance over previously published studies? Please explain.**

Not in its present form (see review)

**Is the paper of broad interest to others in the field, or of outstanding interest to a broad audience of biologists?**

No

**Comments to author:**

The manuscript by Choulet and colleagues presents the annotation and a first analysis of the TE content of wheat and its three different subgenomes. This represents a huge amount of information that should be of interests for scientists working on plant genome structure and TE dynamics. Unfortunately the work presented lacks focus and depth and contains many unsupported conclusions raised from correlations that the authors transform in causal relationships. In my opinion, the manuscript does not contain enough solid and well discussed results to be of general interest and be suitable for publication in Genome Biology.

Major issues

It is not possible to evaluate what part of the TE analysis is included in the general article on the wheat genome referred to as under review (I'm not sure whether the way the authors refer to it -as Science 2018, under review- is appropriate or follows the guidelines of the journal, but this is a different point), and therefore what would be truly original here. The methods and quality of the genome assembly may also impact on the TE prediction, in particular in regions difficult to assemble such as centromeres, that the authors discuss at length here. The authors refer to this article in several places of the manuscript when discussing their results, whereas it is just not possible to get access to this information. For these different reasons, the authors should wait until the genome paper is accessible or otherwise provide it for evaluation.

The authors state that there is almost no sequence conservation of individual TE copies (page 8 line 20). I'm not sure what this means exactly as TEs of the three subgenomes are grouped in the same families which in general have the same abundance. What does exactly means to share sequence conservation?

The authors state that the difference in sequence of individual TE copies (?) is the result of rounds of TE amplification followed by removal of TEs. This should be analyzed in more detail. What is the age of the TEs found in the three subgenomes, are these ages compatible with a recent activity? In fact, an important part of the manuscript is build on the assumption that TEs have maintained an important transposition activity after the split of the three subgenomes from their ancestor, but this is not sufficiently analyzed and discussed.

Related to this, the authors state that there is a high conservation of homeologs gene distance with massive turnover of TEs. Here again more details should be provided to allow a proper discussion. How many new insertions are there in gene regions? What does "massive" exactly means? What is conserved? It does not seem that obvious from the figures and may require a statistical analysis.

The authors deduce from this that chromatin structure is likely under selective pressure. This statement is in my view unsupported. The authors do not provide data addressing this possibility. The hypothesis that (the maintenance of a particular distribution of) meiotic recombination is the reason for the maintenance of the chromosome structure is equally unsupported.

The authors strongly state that the results presented disagree with the accepted idea that retrotransposons (and not TEs at large as stated in the text) amplify in genomes alternating burst of transposition and more quiescent periods. I don't see why the absence of important differences on TE composition (even at the family level) among the three subgenomes can contradict previous results in other genomes. In any case, any discussion on the dynamics of these TEs should be substantiated with phylogenetic analyses.

The section "Presence of genes had..." is vague and full of unsupported conclusions. The title itself can be seen as too trivial (as it is obvious that the presence of genes limits the possibility for neutral TE insertions) or as completely unsupported by the results (if the authors propose an impact that goes beyond what is obvious).

The authors deduce causal relationships from simple correlations (the presence of TEs along the genome is driven either positively or negatively by the presence of genes), and make strong conclusions (the chromatin structure is under selection) that are unsupported by the results.

Additional points:

page 5 lines 10-20. There is no indication of what genome has been used. This should be briefly described here. In the methods section this should also be more clearly defined.

page 5 lines 41-51. The authors first state that half of the difference is due to low copy DNA rather than TE, but then they say that this corresponds to un-annotated sequences that may even be uncharacterized TEs (and presumably repetitive?), which seems contradictory. Have the authors checked that this is indeed low copy DNA?

page 5, lines 53-56. According to table 1, Gypsy elements represent 50.9% of genome A, 46.8% of B and 41.4% of D. Not clear to me why the authors consider 50.9 and 46.8 as similar and 41.4 as lower. Not sure whether statistics is needed, but as it is seems inconsistent. This also impacts the discussion that follows on the top of page 6.

page 7, line 35. "competing for the centromeric niche" implies that the centromeric space is limited and that the success of one family limits the possibilities of the other to insert. This is just one possibility which has not been addressed by the authors. Therefore, this statement lacks support and should be removed.

page 8 lines 47-49. The larger size of the B genome cannot be the consequence of the higher diversity of TE families. The TE population could be highly diverse and occupy the same fraction of the genome if proliferation would be accompanied by elimination. Please revise.

page 11, lines 15-18. "The largest...". What does "the largest" exactly means? The sentence is too general to be accurate. For example, LTR-RTs occupy the majority of the maize genome but not that of Arabidopsis.

page 11 lines 55. Not clear to me why having two times more Copia than Gypsy elements would be an indicator of a younger age of Copia elements.

page 12 line 22. 100% identity of the LTRs does not indicate age 0.

page 12 lines 17-32. This statement is too simple and general. Differential removal of TEs can explain the distribution of some TEs and may help to shape the distribution of others. However, it is well established that some LTR-RTs have insertion biases. The authors actually show that some Gypsy elements are specifically found in the centromere and hypothesize that this is due to their insertion specificity in these regions. In fact the authors acknowledge this in the next paragraph...

page 13, line 54. What does "different subfamilies...that... did not massively exchange" means?

page 14. lines 54-59. It is not clear to me how the data presented will help to characterize the factors that determine the activity of a TE family. Please explain.

page 18 lines 46-51. Again, it cannot be stated that amplification of TEs compensates their deletion without a proper analysis of both phenomena. What does "the intrinsic affinity of a TE with genes" exactly means?

**Reviewer 2**

**Are the methods appropriate to the aims of the study, are they well described, and are necessary controls included?**

Yes

**Are the conclusions adequately supported by the data shown?**

Yes

**Are sufficient details provided to allow replication and comparison with related analyses that may have been performed?**

Yes

**Does the work represent a significant advance over previously published studies? Please explain.**

No, as present in my first comment, at least two reference referred there should be cited and carefully compared.

**Is the paper of broad interest to others in the field, or of outstanding interest to a broad audience of biologists?**

No

**Comments to Author**

Thomas and co-authors analyzed the impact of TEs on the structure and evolution of wheat genome from varous aspects, such as their copies, compositions, sizes, chromosomal distributions, as well as compare them among three sub-genomes of wheat. Their data indicate some interesting explainations, but they are speculates, or discussions, but not conclusions. Below concerns should be addressed.

1) Authors provided data on that there is no association between surrounding TEs and gene expression, and most homoeologous genes keep the same expression patterns. However, Saurabh, et al. found that 98% of paralogous genes were subfunctionized in the manner of expression in maize genome (Saurabh, 2015, MBE). Furthermore, Li et al. observed that TE targeted sRNA was associated to the repressed expression of D subgenome homeologs in wheat (Li, 2014, Plant Cell), which is different to your findings. This should be compared and clarified. I suggest authors to compare these datasets between maize and wheat, and between similar studies of different findings, to finally demonstrate these differences carefully.

2) The abstract only provided general ideas, which is of limit information. I suggest authors to provide more results that have been draw in the main text. Otherwise, readers may lost their interests to read the main text after the first glance of the abstract.

**Authors’ response to reviewers**

***Reviewer #1***:

*The manuscript by Choulet and colleagues presents the annotation and a first analysis of the TE content of wheat and its three different subgenomes. This represents a huge amount of information that should be of interests for scientists working on plant genome structure and TE dynamics. Unfortunately the work presented lacks focus and depth and contains many unsupported conclusions raised from correlations that the authors transform in causal relationships. In my opinion, the manuscript does not contain enough solid and well discussed results to be of general interest and be suitable for publication in Genome Biology.*

*Major issues*

*It is not possible to evaluate what part of the TE analysis is included in the general article on the wheat genome referred to as under review (I'm not sure whether the way the authors refer to it -as Science 2018, under review- is appropriate or follows the guidelines of the journal, but this is a different point), and therefore what would be truly original here. The methods and quality of the genome assembly may also impact on the TE prediction, in particular in regions difficult to assemble such as centromeres, that the authors discuss at length here. The authors refer to this article in several places of the manuscript when discussing their results, whereas it is just not possible to get access to this information. For these different reasons, the authors should wait until the genome paper is accessible or otherwise provide it for evaluation.*

\## Answer 1: We understand this was a problem for the review. Please find a copy of the IWGSC RefSeq manuscript currently under revision in Science (as an attached file of the revised submission). In fact, the IWGSC coordinated a series of articles that are all related to the production of a reference sequence (RefSeq) for the bread wheat genome. Our manuscript about TEs is one of the companion papers. Our manuscript provides an in-depth analysis of wheat TEs that was only briefly summarized in the IWGSC RefSeq manuscript with the reference: "A more detailed analysis of the TE content and its impact on the evolution of the wheat genome is presented elsewhere (Wicker et al. xxx)". This has been coordinated by the editors of the different journals and thus should not be in conflict with the journal guidelines.

*The authors state that there is almost no sequence conservation of individual TE copies (page 8 line 20). I'm not sure what this means exactly as TEs of the three subgenomes are grouped in the same families which in general have the same abundance. What does exactly means to share sequence conservation?*

\## Answer 2: The unclear sentence refers to Fig.2A where we show that in homeologous loci (A vs B vs D), the sequences that can be aligned are restricted to the genes (i.e. homeologous triplets), showing the absence of TE synteny. It means that TEs in the intergenic space have inserted only after the A-B-D divergence while ancestral TEs (i.e. present in the common ancestor) have practically all been removed over time (a process called the "TE turnover" or "genomic turnover"). Non-specific alignments between TEs belonging to the same family may occur (TE homology but not orthology). Since this was unclear, we emphasize now more clearly that sequence conservation is limited to gene space and added a more extensive explanation of the concept of genomic turnover in the revised version (page 8, bottom/page 9, top).

*The authors state that the difference in sequence of individual TE copies (?) is the result of rounds of TE amplification followed by removal of TEs. This should be analyzed in more detail. What is the age of the TEs found in the three subgenomes, are these ages compatible with a recent activity? In fact, an important part of the manuscript is built on the assumption that TEs have maintained an important transposition activity after the split of the three subgenomes from their ancestor, but this is not sufficiently analyzed and discussed.*

\## Answer 3: We agree that dating TE insertions (estimated by LTR divergence) is very important here. We have, in fact, analyzed the age of the LTR-RTs in detail on pages 11 to 14 (section " Dynamics of LTR-retrotransposons from the diploid ancestors to the hexaploid"). As shown in Fig. 4A, most LTR-RTs inserted between 2.5 and 0.5 Mya, i.e. after the divergence of A-B-D genome donors. We added a statement emphasizing this point more explicitly (Page 13, 1st paragraph). We also added more detailed clarifications (Pages 12 and 13).

*Related to this, the authors state that there is a high conservation of homeologs gene distance with massive turnover of TEs. Here again more details should be provided to allow a proper discussion. How many new insertions are there in gene regions? What does "massive" exactly means? What is conserved? It does not seem that obvious from the figures and may require a statistical analysis.*

\## Answer 4: As described above, ancestral intergenic sequences were removed over time and replaced by TEs that have inserted more recently (TE turnover). Thus, intergenic regions are comprised practically exclusively of "new" (i.e. subgenomespecific) insertions. We added this explanation to the beginning of the paragraph and replaced "massive" with the more accurate "near-complete" (page 16). Since practically no TE sequence is orthologous between subgenomes, statistical analysis is prevented. However, we did not report on the statistical test we had performed which showed that actual and randomized data are indeed significantly different. We have added that information to Results (page 16, 2nd paragraph) and Methods (page 26, bottom/27, top).

*The authors deduce from this that chromatin structure is likely under selective pressure. This statement is in my view unsupported. The authors do not provide data addressing this possibility. The hypothesis that (the maintenance of a particular distribution of) meiotic recombination is the reason for the maintenance of the chromosome structure is equally unsupported.*

\## Answer 5: We agree that there is no experimental data to support this conclusion, and that the term "overall chromatin structure" is too broad. However, we believe that our data indicates that distances between genes are conserved by selection. We state this now accordingly (page 16, 2nd paragraph). In our view, these are unexpected and exiting results. Indeed, our data show that, although intergenic regions show no conservation due to TE turnover, the sizes of intergenic regions are conserved (compared to randomized gene positions). Thus, we wanted to provide the hypothesis that the distance between genes, not the intergenic sequence, is under selection pressure. We state it now clearly that this is a hypothesis (page 17, 1st paragraph).

*The authors strongly state that the results presented disagree with the accepted idea that retrotransposons (and not TEs at large as stated in the text) amplify in genomes alternating burst of transposition and more quiescent periods. I don't see why the absence of important differences on TE composition (even at the family level) among the three subgenomes can contradict previous results in other genomes. In any case, any discussion on the dynamics of these TEs should be substantiated with phylogenetic analyses.*

\## Answer 6: The processes at the origin of genome size variation in plants (polyploidy, TE amplification, and deletions) are well known (see Bennetzen et al. 2005 for review). As described above (Answers 2-4), this genomic turnover is a rapid process explaining the lack of intergenic sequence conservation between. Studies in wheat previously suggested that LTR-RTs amplified through bursts followed by rapid silencing (Charles et al. Genetics 2008; Choulet et al. Plant Cell 2010; Daron et al. Genom Biol 2014, etc.). This model of evolution implies that, if TEs evolved by bursts independently in A, B, and D diploid lineages, the 3 subgenomes did not experienced the same bursts, qualitatively and quantitatively, leading to three subgenomes with strongly differing TE compositions. Such independent evolutionary history is supported by LTR-RT age distributions (see answer 3)and explains the observed sub-genome specificities at the subfamily level. But surprisingly, we show that most TE families are present in similar proportions. Thus, despite their independent evolution, TE families amplified at a very similar rate in the 3 lineages, likely maintaining an equilibrium. In addition, we estimated a maximal amplification rate of 600 copies per 10,000 years, which mitigates the "burst" analogy. To address the reviewer's concern, we removed the term "disagree" and rather concluded that our results give more insights into the TE dynamics in large genomes (page 10, last paragraph).

*The section "Presence of genes had..." is vague and full of unsupported conclusions. The title itself can be seen as too trivial (as it is obvious that the presence of genes limits the possibility for neutral TE insertions) or as completely unsupported by the results (if the authors propose an impact that goes beyond what is obvious). The authors deduce causal relationships from simple correlations (the presence of TEs along the genome is driven either positively or negatively by the presence of genes), and make strong conclusions (the chromatin structure is under selection) that are unsupported by the results.*

\## Answer 7: We have taken this remark into consideration and have strongly revised the paragraph in order to make it shorter (reduced by 1/3), more focused on novel results, and without making hypotheses on the evolutionary forces that led to this particular genome organization (Page 17-18).

Additional points:

*page 5 lines 10-20. There is no indication of what genome has been used. This should be briefly described here. In the methods section this should also be more clearly defined.*

\## Answer 8: TEs previously annotated belong to all three subgenomes, with a particular effort made on chromosome 3B (see Choulet et al. Plant Cell 2010). To provide more details, we have added the following sentence in the Results: "…which includes TREP [19]. This represents 3050 manually annotated and curated TEs carried by the three subgenomes and mainly identified on BAC sequences obtained during map-based cloning or survey sequencing projects, especially on chromosome 3B [20]." (Page 5). We also added these details in the Method section.

*page 5 lines 41-51. The authors first state that half of the difference is due to low copy DNA rather than TE, but then they say that this corresponds to un-annotated sequences that may even be uncharacterized TEs (and presumably repetitive?), which seems contradictory. Have the authors checked that this is indeed low copy DNA?*

\## Answer 9: We agree, this statement needs some clarification. The B subgenome is larger than A by 245 Mb, but contrary to what is usually expected (TEs being the main factor at the origin of genome size differences), here we would like to mention that half of this difference is not due to known TEs but rather to the low-copy DNA (in white in Fig. 1A). Since the number of genes is very conserved between A-B-D, this difference is, thus, due to the un-annotated portion of the genome that may correspond to degenerated and unknown non-repeated elements. We revised this paragraph in response to this comment and the following one (page 5/6).

*page 5, lines 53-56. According to table 1, Gypsy elements represent 50.9% of genome A, 46.8% of B and 41.4% of D. Not clear to me why the authors consider 50.9 and 46.8 as similar and 41.4 as lower. Not sure whether statistics is needed, but as it is seems inconsistent. This also impacts the discussion that follows on the top of page 6.*

\## Answer 10: The fraction (in %) of TEs is very similar between A-B-D (83-85%, Fig 1A). This raises the question, what can explain the smaller size of the D genome compared to A/B: ~4 Gb (D) versus ~5 Gb (A/B)? Interestingly, although the D genome is 20% smaller than A and B, this reduction does not imply to all TE superfamilies. In contrast, it appears that Gypsy is the major contributor for this difference: among the 1 Gb difference, 800 Mb is due to Gypsy. However, we agree this paragraph and the conclusion that follows needed revisions and we have therefore revised it in order to avoid inconsistencies (Page 5/6, see also the previous answer).

*page 7, line 35. "competing for the centromeric niche" implies that the centromeric space is limited and that the success of one family limits the possibilities of the other to insert. This is just one possibility which has not been addressed by the authors. Therefore, this statement lacks support and should be removed.*

\## Answer 11: We removed the statement, and added a toned-down version later in the paragraph (Page 7, bottom/8, top).

*page 8 lines 47-49. The larger size of the B genome cannot be the consequence of the higher diversity of TE families. The TE population could be highly diverse and occupy the same fraction of the genome if proliferation would be accompanied by elimination. Please revise.*

\## Answer 12. We agree the sentence was misleading. Kmer analysis revealed a slight difference between the subgenomes: highly repeated kmers cover a smaller fraction of the B genome than A and D. However, B is larger than the others. So, we conclude that the larger size of the B genome cannot be explained by an increase in copy number of highly repeated TEs. We revised the text by clarifying that the B genome contains a smaller proportion of high copy number perfect repeats (Page 9, 2nd paragraph).

*page 11, lines 15-18. "The largest...". What does "the largest" exactly means? The sentence is too general to be accurate. For example, LTR-RTs occupy the majority of the maize genome but not that of Arabidopsis.*

\## Answer 13: The reviewer is correct. We have modified the statement accordingly: "The largest portion of plant genomes with size over 1 Gb consists of long terminalrepeat retrotransposons (LTR-RTs)."(page 10, top).

*page 11 lines 55. Not clear to me why having two times more Copia than Gypsy elements would be an indicator of a younger age of Copia elements.*

\## Answer 14: The flLTR-RTs represent a dataset of intact elements i.e. having both LTRs complete.Therefore, they usually represent young elements compared to old truncated/fragmented copies. Although Gypsy account for ~3 times more DNA than Copia, there are 2 times more Copia than Gypsy that are still intact. This is why we concluded to a younger age for Copia. We modified the sentence to make it easier to follow (page 12).

*page 12 line 22. 100% identity of the LTRs does not indicate age 0.*

\## Answer 15: Indeed, there is a lag period until the first mutations occur, the second smallest age after 0 is 19,000 years. We removed the "age 0" from the text.

*page 12 lines 17-32. This statement is too simple and general. Differential removal of TEs can explain the distribution of some TEs and may help to shape the distribution of others. However, it is well established that some LTR-RTs have insertion biases. The authors actually show that some Gypsy elements are specifically found in the centromere and hypothesize that this is due to their insertion specificity in these regions. In fact the authors acknowledge this in the next paragraph...*

\## Answer 16: We agree there are clearly families that exhibit insertion specificity, especially centromeric elements. Here, the question was: what is the process behind the lower TE density in the distal regions? Is it because TE transpose more frequently into the central regions, or because they are deleted more rapidly from the distal regions? This question was partially answered (based on data for 1 chromosome) by Daron et al. (Genom. Biol. 2014). Here, we confirm these results by showing that young insertions indeed occurred at the same rate in the distal and central regions, confirming that this organization would be due to a higher rate of TE removal in the distal regions.

*page 13, line 54. What does "different subfamilies...that... did not massively exchange" means?*

\## Answer 17: TE families that were present in the A-B-D common ancestor, evolved independently in the 3 diploid lineages, for a few million years, to give birth to different subfamilies i.e., different monophyletic groups on a family tree or different "90/90" clusters. What we show here is that subfamilies are generally specific to one subgenome although we study a polyploid genome. This means that subfamilies amplified in the diploids but not ("massively") after polyploidization, otherwise we would have observed a complete mix between A-B-D copies on the trees at Fig. 5. We modified the sentence as follow: "They evolved to give birth to different subfamilies that, generally, did not massively amplify after polyploidization and, thus, are specific to one subgenome." (Page 14, bottom/15, top).

*page 14. lines 54-59. It is not clear to me how the data presented will help to characterize the factors that determine the activity of a TE family. Please explain.*

\## Answer 18: We now have phylogenetic trees in hands for several hundreds of LTRRT subfamilies representing their individual proliferation lifespans. For example, we can take a closer look at the characteristics of the founder elements to gain insights into common patterns which could explain how and why amplification starts. Elements that do not amplify (at the leaves of the subtrees) could point us to common types of detrimental mutations (related to epigenetic silencing?) which might put a stop to the activity of a lineage. We have added these explanations at the top of page 16.

*page 18 lines 46-51. Again, it cannot be stated that amplification of TEs compensates their deletion without a proper analysis of both phenomena. What does "the intrinsic affinity of a TE with genes" exactly means?*

\## Answer 19: The TE space is not conserved between A-B-D homeologous loci which demonstrates that there was a near-complete TE turnover since these three lineages diverged (~3 Mya; as described above). However, the three subgenomes still contain several Gb of TEs (4.3 Gb for A/B, 3.3 Gb for D). Thus, we can conclude that TE amplification globally compensated their loss by deletion, explaining why the genome size stayed around 4-5 Gb. These forces driving genome size variation were reviewed in Bennetzen 2005, "Transposable elements, gene creation and genome rearrangement in flowering plants", Curr Opin Genet Dev 15(6). To make the message clearer, we modified this paragraph by adding this reference and by removing the expression "affinity with genes" which can be misleading (Page 20, bottom/21, top).

**Reviewer #2:**

*Thomas and co-authors analyzed the impact of TEs on the structure and evolution of wheat genome from varous aspects, such as their copies, compositions, sizes, chromosomal distributions, as well as compare them among three sub-genomes of wheat. Their data indicate some interesting explainations, but they are speculates, or discussions, but not conclusions. Below concerns should be addressed.*

1) *Authors provided data on that there is no association between surrounding TEs and gene expression, and most homoeologous genes keep the same expression patterns. However, Saurabh, et al. found that 98% of paralogous genes were subfunctionized in the manner of expression in maize genome (Saurabh, 2015, MBE). Furthermore, Li et al. observed that TE targeted sRNA was associated to the repressed expression of D subgenome homeologs in wheat (Li, 2014, Plant Cell), which is different to your findings. This should be compared and clarified. I suggest authors to compare these datasets between maize and wheat, and between similar studies of different findings, to finally demonstrate these differences carefully.*

\## Answer 20: We thank the reviewer for interesting discussions. The fact that many homeologous genes kept a similar expression profile is now well established. First, based on the draft genome sequence (IWGSC Science 2014), it was estimated that 79% of homoeologous triads have similar expression pattern, contrasting with the situation observed in other (older) polyploid species like maize. This year, gene expression analysis based on 850 RNASeq samples (32 tissues) and on the reference genome sequence (IWGSC RefSeq manuscript under review in Science) led to the estimation of 84% of the homeologous triads sharing similar expression pattern. Maize is a much older polyploid (~10 Myrs), so that many duplicated genes were lost since this event. What was shown in Pophaly et al MBE 2015 (mentioned by reviewer#2) is that the paralogs that have been retained as duplicates, have a different expression pattern. This difference between wheat and maize can be explained by the date of polyploidization. We have added this discussion in the revised version at page 23/23. In Li et al (Plant Cell, 2014), the authors used newly created hexaploid wheat and observed a repression of genes carried by the D genome compared to the parental allele, repression associated with silencing of flanking TEs. Our results are not in contradiction with this. We just raised another question: are there any TE families particularly associated with genes showing a recent modification of their expression? We modified the title of the section to "No strong association between gene expression and particular TE families in promoters", we have integrated these discussions and comparisons at pages 21-22, and we have included these references.

2) *The abstract only provided general ideas, which is of limit information. I suggest authors to provide more results that have been draw in the main text. Otherwise, readers may lost their interests to read the main text after the first glance of the abstract.*

*## Answer 21: We have revised the abstract, providing more specific results.*

**Second round of Review**

**Reviewer 2:**

**Please provide your comments for the author(s) on the revised manuscript.**

The authors have answered my questions, and I'm satisfied.

The findings of this work are interesting and should be of value to the TE and genome evolution studies.

**Evaluation. Has the author satisfactorily responded to your previous review?**

Yes

On concerning of answers to questions raised by reviewer 1, from my opinion, these authors have made corresponding revisions by removing inaccuracy, confused sentences, or overstated conclusions. They have answered the questions of reviewer 1 properly.

Considering this is an analysis and describtive manusrcipt on general features of TEs in the wheat genome, the current writings as a mixture of conclusions/clues and discussions should be OK.
